# Action Planning for Daily Mouth Care in Long-Term Care: The Brushing Up on Mouth Care Project

**DOI:** 10.1155/2012/368356

**Published:** 2012-04-05

**Authors:** Mary E. McNally, Ruth Martin-Misener, Christopher C. L. Wyatt, Karen P. McNeil, Sandra J. Crowell, Debora C. Matthews, Joanne B. Clovis

**Affiliations:** ^1^Faculty of Dentistry, Dalhousie University, P.O. Box 15000, Halifax, NS, Canada B3H 4R2; ^2^Atlantic Health Promotion Research Centre, Dalhousie University, P.O. Box 15000, Halifax, NS, Canada B3H 4R2; ^3^School of Nursing, Faculty of Health Professions, Dalhousie University, P.O. Box 15000, Halifax, NS, Canada B3H 4R2; ^4^Faculty of Dentistry, University of British Columbia, 2329 West Mall, Vancouver, BC, Canada V6T 1Z4; ^5^School of Dental Hygiene, Faculty of Dentistry, Dalhousie University, P.O. Box 15000, Halifax, NS, Canada B3H 4R2

## Abstract

Research focusing on the introduction of daily mouth care programs for dependent older adults in long-term care has met with limited success. There is a need for greater awareness about the importance of oral health, more education for those providing oral care, and organizational structures that provide policy and administrative support for daily mouth care. The purpose of this paper is to describe the establishment of an oral care action plan for long-term care using an interdisciplinary collaborative approach. *Methods*. Elements of a program planning cycle that includes assessment, planning, implementation, and evaluation guided this work and are described in this paper. Findings associated with assessment and planning are detailed. Assessment involved exploration of internal and external factors influencing oral care in long-term care and included document review, focus groups and one-on-one interviews with end-users. The planning phase brought care providers, stakeholders, and researchers together to design a set of actions to integrate oral care into the organizational policy and practice of the research settings. *Findings*. The establishment of a meaningful and productive collaboration was beneficial for developing realistic goals, understanding context and institutional culture, creating actions suitable and applicable for end-users, and laying a foundation for broader networking with relevant stakeholders and health policy makers.

## 1. Introduction

The last half-century has seen considerable improvements for dental health. Unlike previous generations, more and more older adults are maintaining their natural teeth into old age [1–3]. This is a welcome trend but results in new patterns of disease that become especially significant for those who are frail and who must depend on others for their personal care and hygiene [4–6]. Mouth care is an integral part of personal care yet it is inadequate [4, 6, 7] and given low priority for residents in long-term care [8, 9].

Poor oral hygiene resulting from inadequate mouth care causes considerable morbidity such as mucosal inflammation, caries (tooth decay), and periodontal disease (bone loss around teeth) [7, 10]. Evidence demonstrating links between dental disease and systemic conditions such as respiratory infections [11, 12], diabetes [13–16], and cardiovascular disease [17] also continues to emerge. Tooth loss, pain, and poorly functioning dentures result in problems chewing [18, 19] which is linked to poor nutrition, low body mass index [20], and involuntary weight loss [21]. Dental diseases and dysfunction impact quality of life are known to diminish the pleasures of eating, speaking and social interactions [12, 22]. Overall, the oral health status of residents in long-term care is poor [7, 10, 23–25] and those with dementia experience even higher rates of oral disease [26].

Oral care for dependent older adults in long-term care is becoming a challenge that is expected to grow in importance as our population ages [9]. Over the past several decades, research focusing on attitudes and behaviors of care providers; education and oral hygiene intervention programs; and more recently, environmental, organizational, or social influences on the delivery of care is shedding new light on the complexity of factors influencing the ability to provide adequate oral care.

Practical barriers to oral care include a perceived lack of time [27–29], inadequate staffing levels [27, 28], lack of readily available oral care equipment [30, 31], resistant behaviors by patients/residents [27, 28, 30, 31], and high care staff turnover rates that undermine oral care education programs [32, 33]. Social barriers include embarrassment and repulsion, lack of care staff confidence in their knowledge or ability to provide care [28, 34], a perception of oral care as invasive to the dignity and privacy of residents [35, 36] or unwanted by the resident [35], and the perception by nurses that oral care is professionally unrewarding [36]. Factors identified to facilitate care staff's ability and/or willingness to provide oral care include availability of oral health equipment [37], influence of and examples set by people seen as influential leaders [37], education and/or demonstration in oral health care procedures [27, 29, 35, 37], adequate time to provide care [29], and believing that oral health and oral care are important [28, 29].

Research involving staff education-based interventions directed toward improving oral health status of long-term care residents demonstrates conflicting results [38–40]. Some studies have demonstrated a decrease in disease indicators [32, 39] while others did not [40]. A similarly designed comprehensive personal mouth care program introduced in multiple facilities found different effects on oral hygiene practices and health status of residents of the different facilities [41]. In fact, structural variables expected to influence quality of care (e.g., on-site services, routines, and resources) explained very little of the difference between effective and ineffective programs [41, 42]. Rather, effectiveness appeared contingent upon the organizational context within facilities, comprised of both programmatic strategies and the organizational culture that supports or inhibits them [42].

Creating effective strategies to address the issue of oral health in long-term care is further complicated because oral health care has traditionally been peripheral to mainstream health considerations. The lack of interaction between dentistry and other domains of health care has fostered isolation in approaches for managing oral health. This has been recognized as a shortcoming and has resulted in a call for an interdisciplinary and collaborative approach to both research and practice [8, 9]. Our research attempts to address these shortcomings.

The purpose of this paper is to describe the experience of developing a meaningful interdisciplinary collaboration and to highlight the processes used to design a comprehensive set of actions to integrate oral care into organizational policy and practice within three long-term care facilities in rural Nova Scotia on the east coast of Canada. A collaborative approach is advantageous because the synergy created by the blending of perspectives, resources, and skills of various participants [43] enables the group to create something that is not attainable by single agents [44]. In this study, an interdisciplinary network of researchers and stakeholders were brought together with administrators and front-line care staff to think about the work in creative and practical ways; develop realistic goals; plan and carry out comprehensive interventions that connect multiple programs, services, and sectors; understand and document the impact of its actions; incorporate the perspectives and priorities of stakeholders including the target population; and communicate how actions will address problems [43].

## 2. Methods

### 2.1. The Collaboration

A common interest was established between the principal investigator (M. E. McNally) and a senior administrator of three long-term care facilities during a provincial oral health policy workshop. Over the course of a year, multiple face-to-face meetings, telephone, and email consultations were undertaken with administrative staff to: discuss the current level of oral care and associated challenges; develop realistic and practical research objectives; establish a formal commitment to the research; clarify roles and expectations; and establish mutual benefits. Through this process, a formal collaboration was established consisting of an interdisciplinary research team in partnership with Health Service Managers and Nurse Managers of the three facilities (i.e., the “site team”). All members of the collaboration were involved in establishing goals and strategies for the project. The Nurse Managers were further involved as direct liaisons to each of the sites and assisted with recruitment and data collection. To help focus the research goals and ongoing knowledge exchange, the site team identified training and resources as desired outcomes.

### 2.2. Research Site

Three long-term care facilities were the sites for this research. They are rurally situated within 1 hour of each other and are within 1-2-hour drive of a metropolitan area. The number of residents per site ranged from 25 to 40, which is typical of the majority of long-term care facilities found in rural settings in the region [45]. The three long-term care facilities are administered under the same health district where there is sharing of resources, budgets, and policy. Examining multiple sites within the same organizational structure was undertaken to provide a deeper understanding of how subcultures influence delivery of care at a micro-organizational level [46].

### 2.3. Study Design

A case study approach was used to explore the individual, organizational, and system factors associated with the integration of oral care in three long-term care settings. The unit of analysis was institutional [47]. The case study method was selected in order to gain a holistic understanding of “how” the development and implementation of actions may be influenced by the cultural systems of action that exist within each setting [48].

This research is consistent with the elements of a program planning cycle that include assessment, planning, implementation, and evaluation [49] of a set of actions to integrate oral care into both organizational policy and daily personal care practice. This paper describes details associated with the first two phases, assessment and action planning. *Assessment* involved an exploration of the internal and external factors that influence the provision of oral care and oral disease prevention and was undertaken through a document review, focus groups, and one-on-one interviews. The *action-planning* phase brought care providers and stakeholders (government representatives, educators, and dental professionals) together for a workshop to design a set of actions to integrate oral care into organizational policy and practice in each of the settings. The action plan is being *implemented* in each of the settings over a 12-month period and experiences of the health care team (front-line care staff and administrators) explored. The process and outcomes associated with the implementation of actions are being *evaluated* and are providing the basis of recommendations to revise organizational policy and oral care practice. The latter two phases of the project will be described in a subsequent manuscript at their completion.

A systematic analysis of multiple forms of evidence was used to enhance understanding of the context and the people within it [47]. Sources of triangulation [48] of data include multiple perspectives among investigators (i.e., dentistry, nursing, medicine, administration, policy decision-makers), multiple methods (i.e., document analysis, focus groups, interviews, journaling), multiple data sources (i.e., personal care providers, administrators, residents, and families), and multiple settings. It was also recognized that the term “oral care” connotes the broad spectrum of oral health care needs associated with both professional and personal care. This research is primarily concerned with the latter, “daily mouth care”, for frail older adults. However, it was recognized that the scope of this work might also overlap with clinical and policy considerations of professional dentistry and public health. The term “oral care” is therefore used to encompass the broader range of considerations in the research.

### 2.4. Data Collection

#### 2.4.1. Document Review

Document review was undertaken to provide an organizational and health system context [50]. Members of the collaboration determined the strategy and breadth of the review. Documents were selected on the basis of their topic relevance [51] and included those expected to inform the provision of oral care for long-term care in the region (e.g., job descriptions, clinical guidelines, health assessment tools, accreditation guidelines). Documents were collected directly from the site team, from government, academia, the health services and community college education sectors, and through internet searches of relevant government and education websites. They were individually read, coded, and organized according to four criteria: (1) general health terminology (that may or may not include oral care), (2) oral care (including terms “oral”, “mouth”, and “dental”), (3) foot care, and (4) wound care. References to foot care and wound care were included to provide a basis for comparing oral care to other aspects of care that may be similarly addressed through relevant documents. Members of the collaboration suggested that it would be useful to examine oral care for consistency with existing and familiar clinical domains that may ultimately provide a useful framework for oral care.

#### 2.4.2. Individual Interviews and Focus Groups

Experiential data were collected using a qualitative approach. This approach is constructivist and interpretivist [52, 53] seeking to distil from personal accounts the experiences and meaning behind oral health care for dependent older adults. Qualitative methods are particularly well suited to finding answers to “what” questions (what are people doing, what does it mean) and “how” questions (how are things done, how is meaning produced) [52]. Ethics approval was obtained from the Nova Scotia Capital District Health Authority (CDHA-RS/2009-033). Members of the site team identified potential participants who were invited by letter and a follow-up telephone call. One-on-one semistructured interviews were held with administrators and health professionals who provide a variety of health services to residents and clients associated with the three facilities. Two focus groups were held in each of the three facilities with (1) personal care providers (i.e., those who provide personal care within their job scope including personal care workers, continuing care assistants, and licensed practical nurses) and (2) residents and family members. Residents who had capacity to provide consent (as determined by the nurse manager) were invited to participate and family members were included to represent experiences of those not able to speak for themselves. One author (K. P. McNeil) facilitated focus groups and interviews.

Semistructured questions for both focus groups and individual interviews were designed to guide and generate discussion to elicit participants' description of practices associated with the provision of oral care, perceptions of barriers and facilitators to oral care in the care settings, attitudes toward oral health and oral health care, and relevant knowledge of formal oral health policies. Participants were also asked for suggestions that may improve or enhance oral care. This approach allowed for structure but was flexible enough for participants to raise issues not anticipated. Focus groups and interviews were audio-recorded and transcribed. Each verbatim interview underwent open coding to identify thematically group-related phrases and patterns arising from the data [54, 55] using HyperRESEARCH Qualitative Analysis Tool (Version 2.8.2).

#### 2.4.3. Action Planning Workshop

A one-day interdisciplinary oral care action-planning workshop was held at a location central to the research sites. Members of the collaboration established workshop goals and identified invitees to ensure a broad range of relevant expertise and experience. The purpose of the workshop was to design a set of actions that would integrate oral care into organizational policy and personal care practices in each of the three long-term care settings. The two key goals were to: (1) identify and prioritize education, training, tools, and program strategies and (2) establish a detailed plan for implementing, tracking, and evaluating both professional and daily personal mouth care delivery. The workshop was facilitated by the authors M. E. McNally and K. P. McNeil.

Forty-six invitees were contacted individually by M. E. McNally or K. P. McNeil. The workshop was attended by 34 participants: clinical researchers from dentistry, dental hygiene, nursing, and medicine (*n* = 7); researchers from health promotion and organizational management (*n* = 4); administrators (*n* = 5); nurse managers (*n* = 2); regulated and unregulated front line care staff (*n* = 5); policy makers in the health and continuing care sectors (*n* = 3); representatives of organized dentistry and dental hygiene (*n* = 3); community college educators (*n* = 2); speech language pathologist (*n* = 1); dietician (*n* = 1); as well as a representative from a seniors' government advisory organization. Findings from the document review and experiential data were presented to provide participants with an understanding of context. A review of current best practices [56] and a model of oral health care in long-term care [57] provided an evidence base to inform workshop discussion. Relevant topics identified through the document review, focus groups, and interviews were prioritized by the collaboration for discussion in one-hour breakout sessions and a discussion guide was developed for each topic (Table 1). Each session included 6–8 participants organized to ensure input from a variety of perspectives and disciplines [58].

Responses to the discussion questions were recorded by individual groups and reported back in a plenary session where further input was gathered from the larger group. At the plenary, the group was asked to consider the following: how to best synthesize the information to create a comprehensive “oral care action plan”, how to best communicate the action plan to end users, and how to determine the biggest indicators of success over the next 18 months. Findings from the workshop were collated and synthesized into a draft “oral care action plan” for integration at each of the three long-term care sites. Strategies for implementing and evaluating actions were finalized as part of this process. Following the workshop, details of the action plan were prepared into a report and relevant materials circulated to the site team for final approval. The principal investigator and research coordinator met with both managers and front line care staff at each of the sites to ensure that the proposed action plan accommodated circumstances unique to each setting.

## 3. Results and Discussion

### 3.1. Document Review

Forty-two internal (*n* = 28) and external (*n* = 14) documents were collected and reviewed. Internal documents included information that was directly applicable to the sites (e.g., mission and values, job descriptions, accreditation standards) and external documents provided information on potentially influential outside factors (e.g., Continuing Care Provincial Policy, Professional Standards of Practice). Overall the document scan revealed a general lack of specific reference to oral health and oral care. Consistent with other findings [42], external documents clearly acknowledged a need for or commitment to whole body health and optimal well-being. However, internal documents reflected more direct activities of practice and referred to general terms such as “personal care”, “assessing all body systems”, or “AM/PM care” without the specific mention of oral care. Where oral care was included (*n* = 10), terms such as “mouth/denture care” or “oral care” were used but not described. Overall, the scan revealed negligible references to oral or mouth care as an explicit domain of personal care. There are no government standards with specific reference to mouth or oral care in long-term care. Similar references and level of detail were provided for foot care (*n* = 13). Conversely, the five documents mentioning wound care included details regarding scopes of practice, an interdisciplinary clinic manual, a health services operational report, and a comprehensive manual developed by the provincial government [59]. Details provided for wound care were not surprising given its recognition as specialized care with established best practices for managing wound pathology [60]. This is informative for oral care where the consequences of unchecked oral disease have similar negative implications for health and quality of life.

### 3.2. Individual Interviews and Focus Groups

Thirteen one-on-one semistructured interviews ranging from 30 to 60 minutes were undertaken with administrators and health professionals who service the three facilities. Participants represented two distinct groups, 5 internal professionals who were involved with the day-to-day care of residents living in long-term care facilities (i.e., long-term care coordinators, nurse managers), and 8 external professionals who provided care to residents but who are not present on a daily basis (i.e., physician, dietitian, physiotherapist, occupational therapist, social worker, nurse practitioner, acute care coordinators). The former group was more involved in addressing and recognizing the needs of residents on a daily basis and their perspectives were very much aligned with those of front-line care staff described hereinafter. External professionals recognized the importance of oral care but generally felt removed from oral care and its implications. Neither group was aware of existing formal policies or supports related to oral care in long-term care. Some concerns raised by external participants centered on relevant health risks for residents. Regarding dysphagia, for example, “…*we certainly have concerns about the people pocketing food, going to bed after lunch and that they could choke on that food*”. Lack of consideration for oral care in routine health assessments undertaken by the various professions was also identified as problematic: *“…in a routine screening, I probably wouldn't ask about teeth unless I noticed something and that is probably not a good thing. It is probably something we should be asking about*”. Both groups acknowledged the importance of daily oral care: “*[oral health is important] for overall general health, for nutrition status, for comfort, for self esteem. It's really important for basic health*” and advocated for more educational opportunities for care staff: “*I think that front line people need more education on oral health… I am not sure it is focused enough in the program they take… There doesn't seem to be a lot of emphasis on oral health and why it's important in what I see in the people who come here to work*”. All interviewees were generally aware of difficulties residents have in accessing professional dental services: “*For a lot of our people, they find they can't do anything. If they do have a problem with their dental or oral health, they can't really afford to do anything about it so they tend to leave it*”.

Focus groups held at each of the three sites included 17 residents and family members (*n* = 8, 3,6) and 14 front-line personal care providers (*n* = 5, 3,6). Sessions averaged 90 and 60 minutes, respectively. Residents and family members expressed feedback about availability of mouth care products and good communication between care staff and residents as being important features of mouth care. There was general satisfaction with care provided by personal care staff. Responses to direct questions about daily brushing and denture care met with positive responses by residents: *“They are a great bunch of girls”*. Even with probing questions about daily hygiene care, residents and family members associated mouth care with professional dental care. There was deep concern about a lack of accessibility to professional services. Current residents who did have complaints about their teeth indicated that they would “*make do*” or “*put up*” with the discomfort: *“I'll put up with my teeth”; “I can't bite with [my dentures] like I used to with some things but I think they'll do me”*. Access to professional services was limited by residents' mobility and funding issues associated with transportation costs to move residents off-site for professional care, costs that must be borne by residents themselves: *“It's really expensive to go to a dentist and get a cap or a filling, or even just to have your dentures fixed because some of the elderly, their dentures are loose and they can't afford a new set of dentures. Like who is going to pay for it?” *(Family member). Living in a rural area seemed to further complicate this issue: *“It's practically a whole day by the time you get to the dentist's and back again… Very draining. I get there but by the time I get home, I'm dead.” *(Resident).

Not surprisingly, the most significant input came from front line care staff most involved with the day-to-day care of residents summarized in Table 2. They generally reported that they feel competent to provide mouth care. However, they identified numerous factors that either hindered or helped with carrying out these tasks that are consistent with other reported findings. For instance, although oral care is included in their personal care training, many felt a repulsion and lack of comfort (fear) when providing mouth care [28, 34]. This was intensified when residents exhibit resistant behavior as a result of dementia [28, 34] disability or indifference to the value of mouth care [35]. Participants acknowledged that the proportion of residents and clients with advanced frailty and dementia-related disease is increasing, placing greater demands for providing care [61]. The number of residents with natural teeth is also increasing and many enter long-term care with very poor oral health [1, 34]. In fact, the oral health status of residents was also seen to be an important factor influencing the quality of care they received especially if poor oral health is accompanied by sensitivity or pain. Constraints of resources and time for completing personal care tasks often leave mouth care low on care staff's list of priorities [31, 34]. Although one of the facilities did have a formal oral care protocol, it was acknowledged that there was little guidance for oral assessments, care planning, and accountability. Along with barriers, key facilitating influences were also identified and are consistent with the earlier findings [28, 34]. The level of residents' functional abilities and a good relationship with their care provider were seen as beneficial. Having a good routine, availability of mouth care products, and sufficient time were also identified as important for facilitating care. With respect to perspectives about education, there was a strong indication that standardized and in-depth oral health education during personal care and nursing training programs would be key to achieving improved and consistent daily oral care. Care staff were generally receptive to “in house” education and training opportunities as well. They suggested that “reminders” and “visuals” (such as those commonly posted for hand-washing) would be useful tools for raising awareness. They were unanimous in expressing a resistance to being monitored daily through check lists saying: “*Tick sheets are definitely not the answer*”. Positive reinforcements, available resources, visual reminders, and education would be more readily accepted by care staff for enhancing mouth care.

Overall these findings provided a unique window into the continuing care environment and direct responsibilities of a range of front-line care staff, managers, and administrators working within the three facilities. This feedback, coupled with findings of the document analysis and input from the collaboration, provided the basis for establishing priorities for the action planning workshop, evaluation of prospective activities arising from the workshop, and planning next steps for introducing an oral care action plan.

### 3.3. Action Planning Workshop

As described previously, this one-day interdisciplinary workshop was held to develop an oral care action plan to be integrated into organizational policy and practice. Following formal presentations, significant contributions of the workshop were obtained through direct feedback from workshop participants' small group discussions. Topics and guiding questions are outlined in Table 1. There was some overlap between topics but feedback was collated into the following summaries.

**Education/Training Required to Strengthen the Delivery of Daily Oral Care**

Education for residents may be important to heighten awareness regarding oral health. Strategies should be fun, with creative delivery. Laminated posters should be placed in residents' washrooms. These posters would be used as a visual reminder and would include information on the importance of proper oral care, steps outlining proper care, and so on. They should be bright and colorful and include a number of pictures. A “train the trainers” approach would be appropriate to enhance sustainability of the action plan. This would involve training nursing staff or a designate who would take a leadership role in providing ongoing oral health care support for personal care providers and other relevant staff within the facilities.

**Planning and Tracking Daily Oral Care Activities (Daily and Professional)**

There is a need to change the built environment to provide appropriate space for oral health (i.e., designated space for oral care). To adequately plan and track oral care activities within long-term care, specific tools and resources were suggested. (i) Oral health kits should be created for each resident including the necessary tools to complete oral care such as toothbrushes, denture brushes, toothpaste, mouth rinses, and a towel to protect dentures in sink. The products in kits would be individualized depending on resident's oral care needs. (ii) Care cards should be developed and color coded according to tooth and denture status (i.e., natural teeth, dentures, partial dentures, no teeth) and would facilitate an individualized oral care plan for each resident. Cards could be used by care providers and, if residents go home for visits, by family members. (iii) A tool to enable personal care providers to conduct oral assessments as a part of providing oral care should be developed. The tool would provide guidance for a visual exam of the mouth and a record of any problems. Care providers would need to be educated on what to look for and recognized as being the “eyes of oral care” within the facility. They need to be involved in decision making about what they will be asked/required to do. (iv) A strategy for including oral care in dysphagia assessment that is performed by the Dysphagia Team should be developed. This would allow for a more formalized system of information sharing. By documenting issues related to oral care, it will increase the likelihood that something will be done about it.

**Special Support Needed to Manage Resistant Residents/Residents with Dementia**

A multidisciplinary approach to care planning is necessary. There is a need to raise the profile of oral care for these residents and look at preexisting tools, daily report sheets, and white boards to improve communication regarding oral care. Whatever is done, it needs to be practical for frontline workers. Role-playing may help to put care providers in the residents' shoes.

**Access to Professional Dental Services**

This is an issue that needs to involve everyone from frontline care staff, senior administration, to government. Funding for service is a key issue. Taking residents to a dentist requires funding for transportation in addition to the cost of service. Bringing a dentist to the residents requires funding for space, equipment, and costs of professional service. Ideally, hygienists could make regular visits to facilities. Oral care could be set up similar to foot care where a mobile unit makes site visits. Some mobile services exist but do not currently travel to the more rural areas. Good communication across silos of continuing care and professional dental services is required to improve resident care and potentially save money in the health care system. The value of professional dental care depends on the personal values of residents and their families. Participants recognized that access to professional dental services was an issue beyond the scope of the workshop and of the three facilities involved in the research.

### 3.4. Action Plan Implementation

The strategic action plan evolving from the workshop included each of the activities identified for action in the workshop. The plan emphasized targeted education and training for administrators, nursing staff, and daily personal care providers. The plan specified that oral health manuals should be developed for each site. The manuals would include education materials, pamphlets, and prepared forms to guide oral assessment, care planning, and intervention/referral documents, detailed work-plans, required oral hygiene products such as toothbrushes, toothpaste, denture care products, as well as individualized oral health toolkits for residents.

Proposed actions were implemented over a 12-month period. The site team liaison (or an appropriate designate) assumed responsibility for coordinating activities associated with the action plan. The research coordinator visited each of the three research sites at 6-week intervals to review progress, to provide support, and to gather data. Proposed hands-on education workshops were provided by qualified research team members at the outset of action plan intervention and during regularly scheduled visits. The site team liaison assumed responsibility for overseeing the care and reinforcing the skills with individual care providers as needed. Relevant research team members and the research coordinator provided ongoing support.

### 3.5. Evaluation of the Oral Care Action Plan

To ensure that proposed methods of collecting data to evaluate the action plan activities were relevant and acceptable, the draft evaluation framework was reviewed and refined by the research team based on feedback at the action-planning workshop (Table 3). According to Thorne et al. [42], success in oral health programs in long-term care is contingent upon effective programmatic strategies (e.g., routine oral hygiene, oral assessment, availability of professional dental services) as well as the organizational culture influencing them (e.g., administrative capacity to support and control a caring environment, the presence of “champions”, organizational values) [42]. Recalling that the unit of analysis for this case study was institutional, the evaluation plan was designed to examine the institutional context and to consider both organizational culture and the programmatic strategies arising from the oral care action plan.

## 4. Conclusions

This paper highlights a variety of important considerations for developing meaningful collaborative and applied intervention research. A defining feature of the “Brushing Up on Mouth Care” project has been an enduring and positive collaboration with end-users. This has required careful attention to ongoing communication. It has also necessitated that frequent project updates and face-to-face meetings are balanced with ensuring that end-users do not become “burned out”. Prior to the launch of this project, the collaboration (members of both the research and project site teams) invested time in getting to know each other and in coming to a common understanding of where the research should go. These early communications created a level of comfort and familiarity enabling all voices to be heard and respected. At the outset, this led to the development of realistic research goals about what could be achieved. It also laid the groundwork for the exploratory and planning phases of the project.

The document scan, focus groups, and interviews all contributed to our understanding of the institutional context and organizational culture influencing the delivery of mouth care. This directly informed the action plan that followed. The document scan revealed significant gaps in policy, education, and clinical standards available to guide oral care in long-term care. Our understanding of influences on the delivery of oral care was further informed through the workshop group discussions involving a broader stakeholder group. Here, mechanisms for addressing gaps were also identified and have become integrated into considerations for our ongoing work. One specific example has been the uptake of the “Brushing Up on Mouth Care” action plan by local community colleges responsible for training relevant entry-level care staff. Engagement with government policy makers, directors of care, educators, health administrators, and a broad spectrum of health professionals has also been fruitful in creating awareness about the need for relevant policy as well as guidelines that consider the interdisciplinary nature of this realm of care. Finally, the creation of an oral care action plan that is suitable and applicable to end-users is benefiting both care staff and those who depend on them for care.

## Figures and Tables

**Table 1:** Action planning workshop break-out session guides.

Prioritized discussion topics	Questions for discussion guide
Education/training required to strengthen delivery of care	*Who needs to be involved?* *What should they be doing (i.e., actions/activities)?*
Planning and tracking oral care activities	*What will be required to make activities possible?* *How will we measure/keep track of activities?*
Special supports to manage residents with dementia	*Who will need to be involved in measuring/tracking progress?*
Access to professional dental services	*How will we know if activities are successful?*

**Table 2:** Personal care providers narrative findings—barriers, facilitators and education.

	Explanation of theme	Supporting quotes
**Barrier themes**		
Repulsion/fear	*Sometimes care providers are repulsed by certain aspects of oral care such as halitosis, or a resident spitting/coughing on them. Care staff are fearful of providing oral care for a variety of reasons (e.g., drop or break dentures, cause the resident to gag or aspirate, get bitten). *	“So I had to clean them; oh it was gross… I don't know how she even handled it but I guess it'd been like that and she had just gotten used to it.” “You have to be careful because if you want to stick your finger in or anything close they can bite you.”

Resident disability/dementia/resistance	*Oral care provision is more complicated when a resident is disabled or has dementia. Often residents cannot express themselves when they are confused or suffering from oral pain or discomfort and this may be interpreted as resistant behaviour.*	“If you have a [resident with] dementia that might have some of their own teeth and can't tell you he's a got a toothache, you know what he's going to do… they're going to act out. …They become agitated and they can't express it.” “Sometimes it's hard to do oral care with people with dementia because they don't want you around their mouth; they don't know exactly what you're doing.” “I mean somebody who's got advanced dementia there's no sense, just work with them and hope for the best.”

Resident attitude/indifference	*Often residents appear to not care or are unaware of the importance of oral health. Many residents would not have gone to the dentist for regular check-ups throughout their lives and therefore oral care is not a priority for them.*	“People years ago didn't go to a dentist unless it really bothered them and they had an abscess and then they went to the family doctor and he gave them antibiotics and then he pulled the tooth out.” “A lot of residents just don't want to be bothered [with oral care]… it's just not something that's important to them.” “I think he wouldn't say a word if you didn't get to his teeth.”

Current oral health status of resident	*If a resident comes into a facility with poor oral health, it is more difficult to provide them with adequate oral care, especially if they have sensitivity, discomfort, or pain. *	“[Resident Name] has very few teeth and has had over the years very poor mouth care, therefore he's got infections in his gums and his teeth are rotten.” “Yes it makes you wonder if they have a bad history their whole life of bad mouth care. And that's why their teeth are so bad, or is this decline just recent, like within the last five years or whatever.”

Lack of time	*“Visible” activities (dressing, combing hair, washing, etc.) take priority when there is a time crunch (e.g., in the morning). Staff indicated that if they had more time, oral care would likely get more attention. *	“If somebody's in a hurry… It's a wham, bam, thank you, ma'am, the teeth can be left.” “I think the people that have their own teeth probably don't get the attention. Now as far as I'm concerned, they need more attention because they have their own teeth, but I think they're the ones that get neglected because of the fact that it takes longer to do natural teeth than it does dentures.”

**Facilitator themes**		
Resident ability	*It can be helpful when residents are aware of their oral care and remind staff to brush their teeth. Having the resident provide the cue often ensures their teeth will be done. *	“We have two [residents] that will actually ask, will you brush my teeth?” “[Name of resident] is very insistent on having her teeth done after breakfast and before she goes to bed and her teeth are done faithfully.”

Resident relationship with care provider	*Having a good relationship with a resident can make oral care provision easier. The care provider is familiar with likes/dislikes and routines and the resident is more comfortable around them. *	“You know what [the residents] want…. they sort of trust you… they feel, they don't care if you see them without their teeth [in].”
Proper tools and products	*Oral care provision is easier when the necessary tools are available and on-hand. Using the proper tools for specific care needs is also important (e.g., denture brushes for dentures, child size toothbrushes for residents with small mouths). *	“Having everything there right where you can get it; you know your toothbrush, toothbrushes and things; just having it right close.” “I wish we had those little toothbrushes back… [they] curved like this, so every time you used them it would get right in around their gums and everything else; it brought a lot of stuff out.”

**Education themes**		
Oral health education	*Additional oral health care training may be beneficial for care providers who are currently in the workforce as well as family members or volunteers.*	“A lot of these [care providers] have been doing this for 25 years, they never took a course and were just grandfathered in… it's really hard for you to get across to them that just because you've been doing that that way doesn't mean you were doing it the right way. So a lot of people figure you're making waves if you say something.” “Sometimes family members need to be educated; and just to be aware of what we're trying to do like promote good oral care; sometimes they say “If mom or dad won't open their mouth then don't make them”. [Then] there's nothing I can do.”

Education tools	*Tools suggested that would be helpful with oral health education and awareness (apart from formal education).*	“Well we have hand-washing posters all over the place, why not oral care posters?” “So if there was posters [about oral care] in each of the elders rooms, in their bathrooms, right by their sink then you're there with the teeth or with the elder, you're going to read it.” “If this could be one of the subjects that is brought up at every care conference, also, every time we do rounds. Now, rounds is for a wing, a whole wing in general, so if oral care could be brought up then and discussed, just like I said, keep it fresh, keep it going, keep it on everybody's mind.”

Care provider training programs	*Care providers should receive standardized, in-depth training on oral care provision.*	“So that's where the education has to come in—that everybody realizes what oral care is and what it entails.” “It's always good when we have new young ones coming in because they're fresh out of the course and they've learned from the book the right way; so I always like to see them coming in.”

**Table 3:** Evaluation Framework.

Outcome variables	Data source	Proposed metrics/indicators
**Programmatic strategies**		
Integration of individualized oral care plan	Focus group and key informant narratives Administrator diary studies Document review (e.g., policy)	Thematic analysis Proportion of residents in whom oral care is discussed during care planning meetings

Oral assessments	Oral care activities records	Summary data

Professional dental care	Oral care activities records	Use of referral systems (e.g., to dentist)

Daily mouth care protocol	Focus group and interview narratives Diary studies Oral care activity records	Thematic analysis Oral care product use

Material indicators of program uptake	Dental supply inventory Dental supply orders	Summary data

Nonmaterial indicators of program uptake (e.g., time allotment formal and informal practices)	Focus group and key informant narratives Diary studies Document review	Thematic analysis

**Organizational culture**		
Behavior/attitudes of staff toward delivery of oral care	Focus group and key informant narratives AWS Care provider and administrator diary studies	Thematic analysis Mean change in AWS scores

Satisfaction/acceptability of staff/residents/families	Focus group narratives and interviews	Pre/postintervention comparison of themes and patterns

Staff knowledge of oral health	Posteducation knowledge uptake questionnaires	Attendance at orientation and education in-service Pre/posteducation knowledge (scores)

Organizational values	Key informant narratives Document review	Pre/postcomparison of themes and patterns
